# Gangrenous Small Bowel Due to Reposition of Procidentia in an Elderly Woman

**DOI:** 10.7759/cureus.25013

**Published:** 2022-05-15

**Authors:** Harshal Tayade, Surekha Tayade, Meenakshi Yeola, Yashwant Lamture

**Affiliations:** 1 General Surgery, Jawaharlal Nehru Medical College, Datta Meghe Institute of Medical Sciences, Wardha, IND; 2 Obstetrics and Gynecology, Jawaharlal Nehru Medical College, Datta Meghe Institute of Medical Sciences, Wardha, IND; 3 General Surgery, All India Institute of Medical Sciences, Mangalagiri, IND

**Keywords:** bowel gangrene due to reposition of prolapse uterus, gangrenous bowel due to pelvic organ prolapse, small bowel gangrene and advanced pop, pelvic organ prolapse (pop), pelvic organ procidentia

## Abstract

Gynecologists are familiar with procidentia, a severe form of pelvic organ prolapse (POP) that includes herniation of the anterior, posterior, and apical compartments of the vagina, through the introitus. Usually, women with POP present with concerns of something coming out of the vagina, heaviness, discharge through the vagina, urinary complaints, and, rarely bowel, complaints. Intestinal obstruction secondary to procidentia is a rare complication and is seldom reported in the literature. We report one such case where an elderly woman presented with the primary concerns of constipation, retention of urine, and multiple episodes of vomiting. Clinical history revealed that herniated tissue protruding outside the vaginal introitus was reposited inside the vagina two days ago. Clinical examination and investigations were suggestive of intestinal obstruction, secondary to the reposition of procidentia. Exploratory laparotomy revealed gangrene of the terminal ileum. Right hemicolectomy with ileo-colic anastomosis was done, which saved the woman’s life. Reposition of the prolapsed uterus was thought to be the probable reason, leading to obstructed and gangrenous small bowel. As this case illustrates, the chronology of symptoms and signs and progression of disease should be appropriately interpreted to diagnose and manage such potentially life-threatening conditions.

## Introduction

Pelvic organ prolapse (POP) is defined as the descent of pelvic organs into the vagina from their normal anatomical position, as a result of the weakness of supportive structures such as ligaments and pelvic fascia [1]. The term ‘Procidentia’ is derived from the Latin word ‘procedere,’ which means to fall. It is an extreme form of POP wherein all the three compartments of the pelvis herniate out of the vaginal introitus [2]. Based on the descent of the compartment, POP is broadly classified as cystocele where anterior compartment herniates, rectocele where posterior compartment herniates, and if apical portion or loops of intestine prolapse through the upper part of the posterior compartment, it is known as enterocele. The symptoms of POP are urinary dysfunction [3], inability to pass urine, stress urinary incontinence, sexual dysfunction, bowel problems like difficulty in defecation, incomplete evacuation, local symptoms like abnormal discharge through the vagina, something coming out of the vagina, and heaviness in the pelvic area. Multiple risk factors have been cited for POP such as ethnicity, multiparity, childbirth-related conditions like early bearing down efforts, a prolonged second stage of labor, menopause, occupational hazards, conditions causing an increase in intra-abdominal pressure, and connective tissue disorders like Ehler Danlos syndrome. Around 50% of parous women have some degree of POP during their lifespan [4]. Among them, 10-20% will be symptomatic [5]. They seek medical care only when the prolapse becomes symptomatic and hinders routine activity. Every woman has an 11% lifetime risk of undergoing POP surgery [6]. Small bowel obstruction is a “mechanical or functional obstruction of the intestines, which prevents the normal movement of the products of digestion” [7]. Clinically, it is diagnosed by a classical triad of obstipation (absolute constipation), abdominal distension, and vomiting. Abdominal pain and distension usually precede the appearance of nausea and vomiting by several hours. Intestinal obstruction or gangrene is a rare complication of procidentia and scarce information is available about it in the literature. Such a case has not been documented in available medical publications. However, once the woman has clinical features of an acute abdominal event and intestinal involvement, the situation converts into an emergency and quick decisions have to be taken to save the patient’s life.

## Case presentation

**Figure 1:**
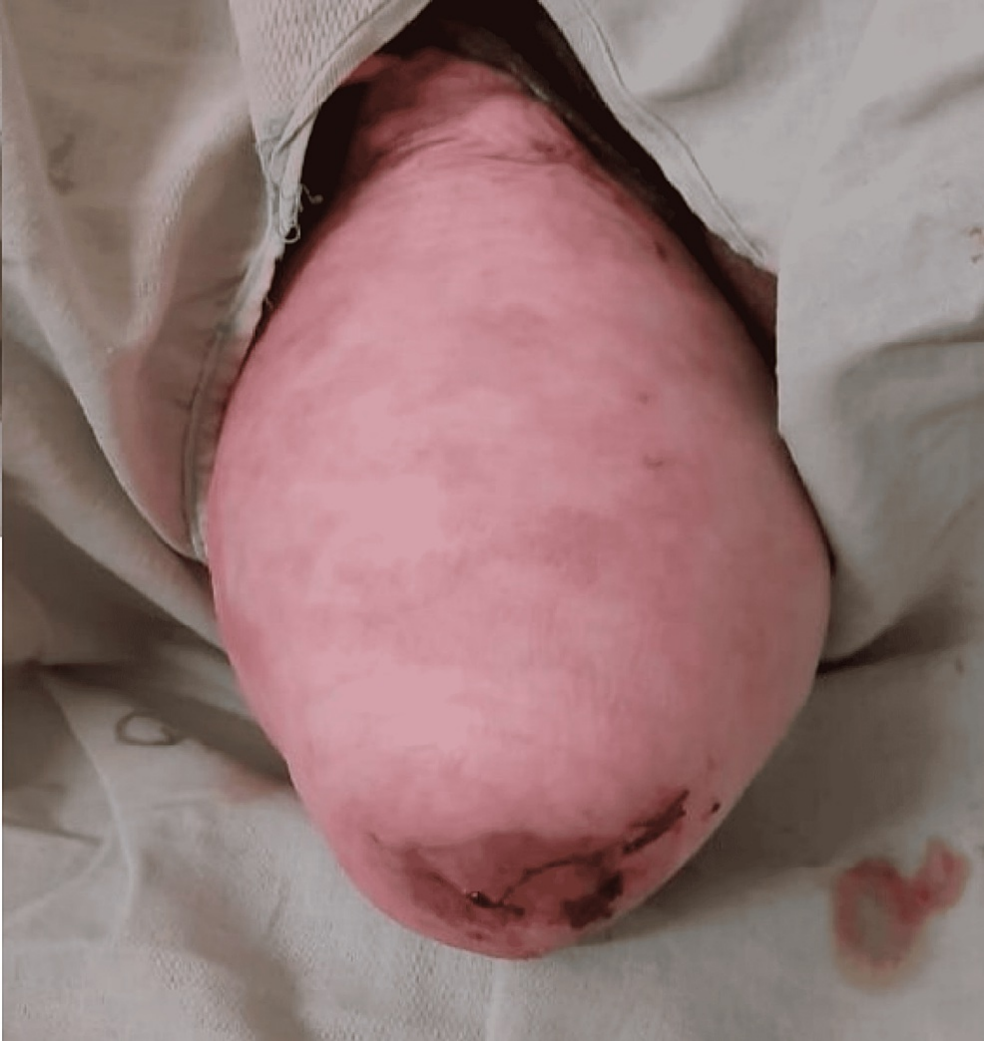
Complete procidentia with herniation of the anterior, apical, and posterior compartment of the pelvis through vaginal introitus; decubitus ulcer is seen on the cervix

A 50-year-old-female, with long-standing, complete procidentia was referred to the surgical unit of a rural hospital in central India during emergency hours. She was a fourth para, having two living children and all four childbirths had taken place vaginally, at home. She was a farmer by occupation and presented to the ED in the month of March 2020 during the period of strict lockdown in India for the coronavirus disease 2019 (COVID-19) pandemic. She had concerns about retention of urine and pain in the abdomen for 24 hours and a history of something coming out of the vaginal introitus for 10 years. Before the acute event, she was relatively all right, and over a period of 12-24 hours, she had retention of urine and associated severe generalized abdominal pain. Thus, she reported to casualty at midnight in spite of the lockdown restrictions and ongoing pandemic. Clinical examination showed that she had third-degree uterine descent, cystocele, rectocele, and eversion of the vagina. A decubitus ulcer was present on the anterior wall of the vagina (Figure 1).

**Figure 2:**
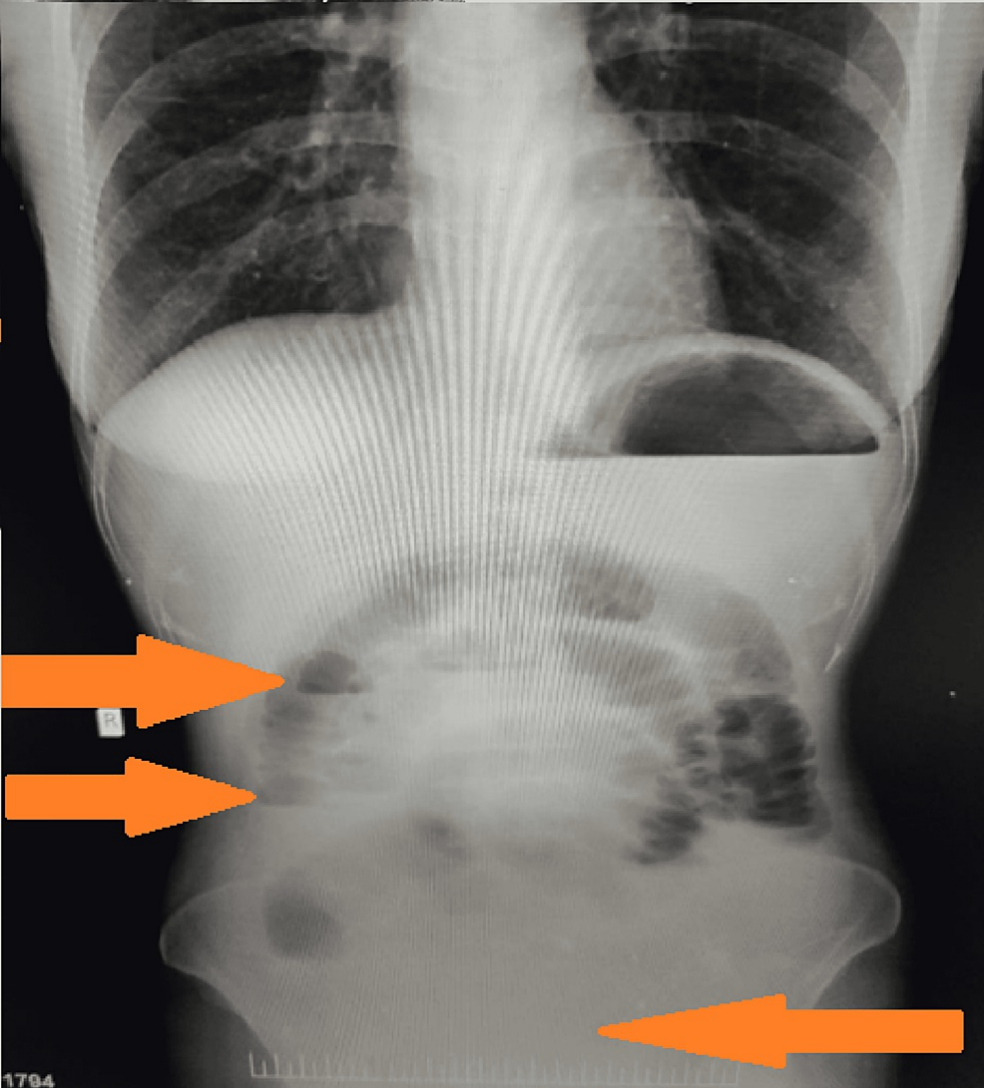
X-ray abdomen (AP erect) showing features of small bowel obstruction (loss of gas in the pelvis and multiple air-fluid levels) (Hazy, as obtained from the emergency department) AP: anteroposterior

During this particular period, a strict lockdown had been imposed throughout India. There was apprehension in the community about getting infected with COVID-19 and hence, this lady was not willing to get admitted to the hospital. As emergency management, to treat retention of urine, the tissue herniating outside the vagina was reposited inside and vaginal packing was done with tape gauze. The woman improved symptomatically, and she went home. Two days after discharge, she again presented to the emergency room with concerns of obstipation and failure of passage of stool and flatus, abdominal pain, distension, and vomiting. She had tachycardia, her pulse was 110 beats per minute, and her blood pressure was 116/78 mm Hg. The abdomen was distended, diffusely tender to touch, and had hyper-resonant notes on percussion. Bowel sounds were sluggish in all four quadrants of the abdomen. A digital rectal examination (DRE) revealed an empty and ballooned distal rectum. The examining finger was unstained with feces. There were centrally located, multiple air-fluid levels, dilated small bowel shadows, and loss of gas in the pelvis on plain X-ray abdomen (Figure 2).

**Figure 3:**
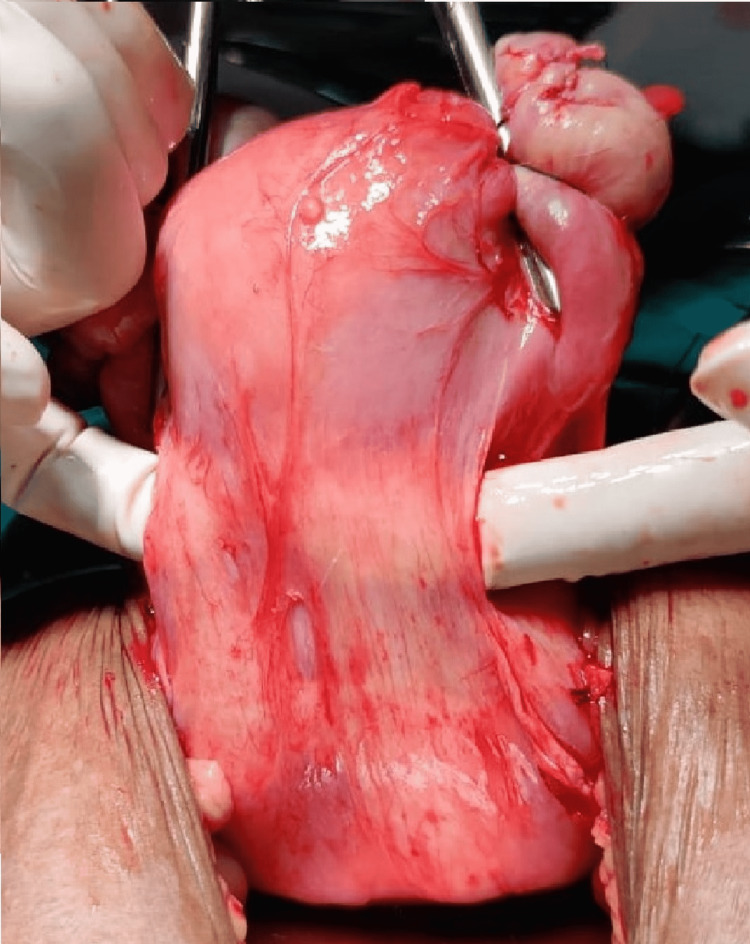
Band of adhesion present posteriorly between the isthmic region of the uterus and rectum

All these features were suggestive of small bowel obstruction. Exploratory laparotomy was performed by a team of surgeons and gynecologists. Intraoperatively, we found that the uterus was enlarged up to 10-12 weeks pregnant uterus size and dense adhesions were present between the rectum and isthmic region of the uterus (Figure 3).

**Figure 4:**
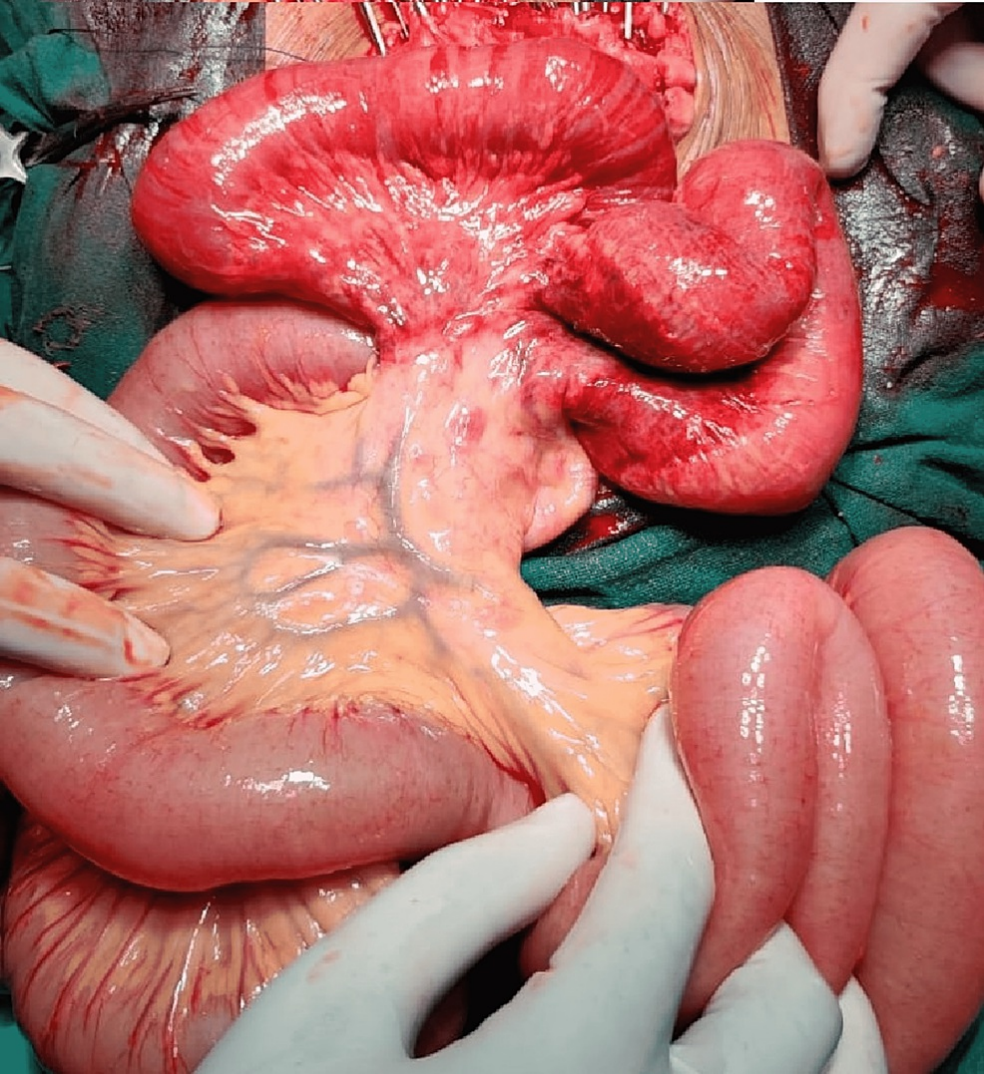
Ischemic and gangrenous small bowel

Adhesiolysis and total abdominal hysterectomy with bilateral salphingo-oophorectomy were performed by careful dissection. The gynecologist performed the hysterectomy and handed the procedure over to the surgical team. A 20 cm length of the small bowel, from 5 cm proximal to the ileocecal junction, was found to be gangrenous (Figure 4).

Due to the vicinity of the gangrenous segment to the ileocecal junction, a right hemicolectomy with ileo-colic anastomosis was done. The woman recovered well postoperatively and was discharged after 10 days. The histopathology report of the excised tissue was suggestive of wet gangrene of the ileum.

## Discussion

POP is a well-tolerated, benign, gynecological problem found in women and has site-specific symptoms. It does not warrant treatment unless it is symptomatic. Procidentia, a chronic condition, is complete herniation of the uterus, out of the vaginal introitus along with anterior and posterior vaginal walls. Rural women often postpone seeking medical assistance in spite of having longstanding procidentia, resulting in further complications. Kumari et al. reported that more than half of the subjects in their study did not obtain any treatment for uterine prolapse and among those who did, some waited more than 10 years before consulting a doctor while many did so only more than one year after the onset of prolapse [8]. Bang et al. also reported similar findings [9].

In the present case too, the woman did not seek medical attention for almost 10 years and presented with acute symptoms of urinary retention. Constipation is the common symptom associated with POP, specifically in posterior compartment prolapse (rectocele). In the present case, there was acute development of constipation, which was associated with episodes of vomiting, which forced the treating team to think of an alternative diagnosis. The findings of this case illustrate the importance of meticulous clinical examination in a common scenario like POP to rule out other underlying serious, surgical conditions like bowel gangrene. Though constipation may be common in POP, full-blown small bowel obstruction is extremely rare. Few cases of small bowel obstruction associated with post-hysterectomy POP and enterocele have been documented in the literature [10-12]. However, this case is unique, as the obstruction was probably precipitated during the reposition of the prolapsed uterus inside the vagina.

## Conclusions

This case illustrates that counseling every patient for post-procedure observation is important, and there should be close monitoring, especially when the bladder or bowel is handled either directly or indirectly. Adequate attention should be rendered to symptoms, their progression, and chronology. High suspicion for “acute abdomen,” with prompt follow-up and rapid and relevant investigations, is recommended in patients presenting in emergency settings. A multidisciplinary approach is imperative in such complicated cases. Procidentia is a long-term disease and many women prefer to seek the opinion of family physicians for minor symptoms related to it. It is suggested that family physicians should be aware of the rare but lethal complications of procidentia so that prompt referral can be done and the associated morbidity and mortality can be reduced.
